# Low sensitivity of the tourniquet test for differential diagnosis of dengue: an analysis of 28,000 trials in patients

**DOI:** 10.1186/s12879-016-1947-7

**Published:** 2016-11-03

**Authors:** Nathália Barbosa Furlan, Caroline Tukasan, Cássia Fernanda Estofolete, Maurício Lacerda Nogueira, Natal Santos da Silva

**Affiliations:** 1 Faculdade de Medicina, União das Faculdades dos Grandes Lagos, São José do Rio Preto, São Paulo Brazil; 2 Laboratório de Pesquisas em Virologia, Faculdade de Medicina de São José do Rio Preto, São Paulo, Brazil; 3 Laboratório de Modelagens Matemática e Estatística em Medicina, União das Faculdades dos Grandes Lagos, São José do Rio Preto, São Paulo Brazil; 4 Laboratório de Modelagens Matemática e Estatística em Medicina, Faculdade de Medicina, União das Faculdades dos Grandes Lagos, Rua Dr. Eduardo Nielsem, 960 Jardim Novo Aeroporto, São Jose do Rio Preto, São Paulo CEP 15030-070 Brazil

**Keywords:** Dengue, Tourniquet test, Diagnostic test for dengue

## Abstract

**Background:**

The aim of this study was to evaluate the utility of the tourniquet test (TT) for dengue diagnosing. To our knowledge, no previous study with such a large sample, of this duration, with as many laboratory methods referenced, or relating the results of the TT to the 2009 WHO classification of severity has been conducted thus far.

**Methods:**

In this study, we analyzed the records of 119,589 suspected dengue cases in a Brazilian city, with 30,670 confirmed cases. The Cohen’s Kappa test was applied to evaluate the degree of agreement between the tests, and the sensitivity and specificity was calculated for the TT.

**Results:**

Twenty-eight thousand six hundred thirty-five TT were performed. No association between the outcome of the TT and greater severity of infection, according to the 2009 guideline, was observed (*P* = 0.28); furthermore, relevant agreement with the final diagnosis (κ = 0.01; 95 % CI = 0.00 to 0.02) or individually with the IgM enzyme-linked immunoassay was not observed (κ = 0.05; 95 % CI = 0.04 to 0.06), and was even lower with PCR (κ = 0.27; 95 % CI = 0.06 to 0.49). Most importance of the TT was shown in relation to specificity (88.9 %; 95 % CI = 0.88 to 0.89) and negative predictive value (70.3 %; CI 95 % = 0.70 to 0.71).

**Conclusions:**

TT was more effective in detecting cases that were truly negative than positive. These results suggest that the TT should not be used as diagnosis of dengue.

## Background

The dengue virus (DENV) is the most important arbovirus in the world in terms of morbidity, mortality, and clinical implications [1]. Between 2.5 and 3.6 billion people, more than 50 % of the world’s population, are currently living with the risk of infection. The incidence is even higher in tropical and subtropical countries where environmental conditions favor the proliferation of the *Aedes* mosquitoes [2–5]. However, this disease is also advancing into developed countries, where the occurrence of dengue has not been previously registered, such as the United States of America [6–8], Japan [9–11], and France [12].

Dengue is caused by four types of DENV: DENV-1, DENV-2, DENV-3, and DENV-4 [13]. More recently, a new serotype, DEN-5, was identified in serum samples collected during an epidemic of dengue in Malaysia in 2007, still under study [14]. All virus strains can lead to all forms of dengue infection, from asymptomatic to severe, or even fatal. However, it is unclear why some dengue cases progress to severe forms. Although there are controversies in the literature, sequential infections of different serotypes of DENV have an important role [15–17]. Still, even during epidemics in populations with high levels of antibodies against DENV, the proportion of severe dengue cases is low [18], suggesting that other risk factors are involved in the severity of the disease.

The main feature of the severe forms of the disease is increased vascular permeability resulting in plasma leakage from the intravascular compartment to the extravascular space [17, 19, 20]. Fluid loss, leading to hypovolemic shock, thrombocytopenia, coagulation abnormalities, and bleeding are also features of severe dengue disease [21]. The diagnosis of the infection is particularly difficult in mild forms of the disease, especially when differentiating dengue from other febrile illnesses. The risk of swift progression from mild to severe forms of dengue infection, which may require rapid resuscitation measures, demonstrates that accurate and early diagnosis through other methods is relevant in the absence of serologic and virologic confirmation [22–24].

In 1997, the World Health Organization (WHO) listed the tourniquet test (TT) as a criterion for dengue hemorrhagic fever, and that the positive test reflects both capillary fragility and thrombocytopenia [22]. Studies suggested that the test has a greater positivity rate in individuals with more severe forms of the disease but cannot exclude dengue infection [25, 26].

Evaluation of the utility of TT to diagnose or exclude dengue has been proposed in previous studies [20, 22, 27, 28], with mixed results. Thus, the aim of this study was to evaluate the use of TT in the screening of patients with suspected dengue and its severity, and to compare this test with routine diagnostic methods. Accordingly, we decided to use a relatively large sample over a considerably long time period. We also utilized many laboratory methods for reference, including the polymerase chain reaction (PCR), and simultaneously related results to the severity index set forth in the 2009 WHO classification.

## Methods

We have used records from the Notifiable Diseases Information System (SINAN), a program of Brazil’s Ministry of Health to collect notifications of all suspected dengue cases in the city of São José do Rio Preto, São Paulo, Brazil, whose population in 2010 was approximately 408,258 inhabitants. For this study, 119,589 cases notified between May 1998 and July 2012 were eligible, previous to the introduction of Zika and chikungunya virus in Brazil.

In addition to clinical evaluation by a physician, serum samples from all patients with suspected dengue were submitted to a diagnostic laboratory for the determination of dengue based on an IgM immunoglobulin enzyme-linked immunoassay (ELISA), non-structural protein 1 (NS1), or DENV PCR, depending on which test was being commonly used at the time of the case.

### Case definition

For notification purposes, in accordance with the criteria of Brazil’s Ministry of Health, suspected cases of dengue included all patients with acute febrile illness lasting up to seven days, accompanied by at least two of the following signs or symptoms: headache, retro-orbital pain, myalgia, arthralgia, prostration or rash associated with the presence of bleeding, positive epidemiological history, residence within a dengue transmission or *Aedes aegypti* endemic area in the last 15 days.

The clinical classification used retrospectively in this study for statistical purposes was based on the WHO guideline (2009) to distinguish between dengue and severe dengue. The first one is suspected in patients who live or have traveled to a dengue-endemic area, with fever and two or more of the following: nausea and/or vomiting, rash, pain (headache, retro-orbital pain, myalgia, and/or arthralgia), and a positive TT or leukopenia. Abdominal pain, persistent vomiting, fluid retention, mucosal bleeding, lethargy, hepatomegaly greater than 2 cm, increasing hematocrit, and rapid decrease in platelet count are suggestive of dengue and serve as warning signs of severe dengue. Severe dengue is characterized by severe plasma leakage leading to shock or respiratory distress, severe bleeding, impairment of organs with a pronounced increase of aminotransferases, altered consciousness level, or heart failure.

### Statistical analysis

All reported cases (negative or positive for DENV) in which the TT was performed were evaluated, and this variable was studied in relation to the actual outcome, i.e., confirmed dengue on the basis of the conventional laboratory methods as described above. For cases in which the diagnosis of dengue was established, the percentage of positive TT results was evaluated over time and according to age.

A descriptive analysis of personal and serological variables was performed and related to a hemorrhagic manifestation as externalized bleeding and thrombocytopenia, defined as a platelet count of less than 100,000/mm^3^.

The hypothesis that TT positivity would be associated with more severe scores of DENV infection was evaluated using nonparametric Mann-Whitney U tests. However, the Pearson’s chi-square test (*χ* ^2^) was performed to check if there was a positive association between TT and thrombocytopenia in patients with suspected dengue. The Cohen’s Kappa coefficient (κ) was applied to measure the degree of agreement between the TT and the final diagnosis, PCR results, and presence of IgM and NS1. To quantify the strength of this agreement, a scale used by Landis and Koch was proposed, where values lower than zero show poor agreement. Specifically, values less than zero indicate poor agreement, from 0 to 0.2 indicate slight agreement, 0.21 to 0.4 indicate fair agreement, 0.41 to 0.6 indicate moderate agreement, 0.61 to 0.8 indicate substantial agreement, and 0.81 to 1.0 indicate almost perfect agreement [29].

Later, the sensitivity and specificity, positive predictive value (PPV), negative predictive value (NPV), positive likelihood ratio, and negative likelihood ratio of the TT were also determined. All analyses were performed using SPSS software, version 19, and adopted a significance level of 5 % and a confidence interval of 95 %.

## Results

We analyzed 119,589 participants, with an average age of 33.3 years, with considerable dispersion (±18.1 years); further, 65,595 (54.9 %) of the patients were female and 53,898 patients (45.1 %) were male (96 patients had no information about sex). The TT was performed in 28,635 patients (23.9 % of all notifications); 3252 (11.4 %) were positive and 25,383 cases (88.6 %) were negative.

At least one information about hemorrhagic manifestation was available for all patients undergoing to TT. The data shows that 17,123 (59.8 %) patients had not shown any hemorrhagic manifestations, while 11,512 (40.2 %) had one or more type of blood leakage (Table 1). However, information about thrombocytopenia was obtained in only 1930 (6.7 %) individuals. In these patients 906 (47.0 % of patients) had negative TT results without thrombocytopenia, 269 (13.9 %) had negative TT results with thrombocytopenia, 538 (27.9 %) had positive TT results without thrombocytopenia, and 217 (11.2 %) had positive TT results with thrombocytopenia. Therefore, there was a statistically significant difference between the TT results and the thrombocytopenia count of these patients (*χ* ^2^ = 8344; *P* = 0.004).

**Table 1:** Hemorrhagic manifestations in patients undergoing tourniquet test

Hemorrhagic manifestation	Tourniquet test			
	Positive	Negative	Total	
	*N* (%)	*N* (%)	*N* (%)	*p*-value
Epistaxis^a^				<0.001
Present	121 (17.5)	571 (82.5)	692 (100)	
Ausente	3032 (11.2)	23,954 (88.8)	26,986 (100)	
Petechiae^b^				<0.001
Present	701 (29.7)	1663 (70.3)	2364 (100)	
Absent	2445 (9,7)	22,694 (90.3)	25,139 (100)	
Exanthema^c^				<0.001
Present	1382 (18.8)	5984 (81.2)	7366 (100)	
Absent	1693 (8.3)	18,762 (91.7)	20,455 (100)	
Gingival bleeding^d^				<0.001
Present	76 (19.7)	309 (80.3)	385 (100)	
Absent	3073 (11.3)	24,181 (88.7)	27,254 (100)	
Metrorrhage^e^				<0.001
Present	34 (27.4)	90 (72.6)	124 (100)	
Absent	1673 (15.2)	9366 (84.8)	11,039 (100)	
Hematuria^f^				0.15
Present	16(18.2)	72(81.8)	88(100)	
Absent	2639(12.9)	17,880(87.1)	20,519(100)	
Gastrointestinal^g^ bleeding				<0.001
Present	33 (25.6)	96 (74.4)	129 (100)	
Absent	2613 (12.8)	17,732 (87.2)	20,345 (100)	

Missing: there was no information for the respective hemorrhagic manifestations

*p*-value: Chi-square

^a^Missing: 957 (3.3 %)

^b^Missing: 1132 (4.0 %)

^c^Missing: 814 (2.8 %)

^d^Missing: 996 (3.5 %)

^e^[Information about female] Missing: 4807(30.1 %)

^f^Missing: 8028 (28.0 %)

^g^Missing: 8161 (28.5 %)

**Fig. 1:**
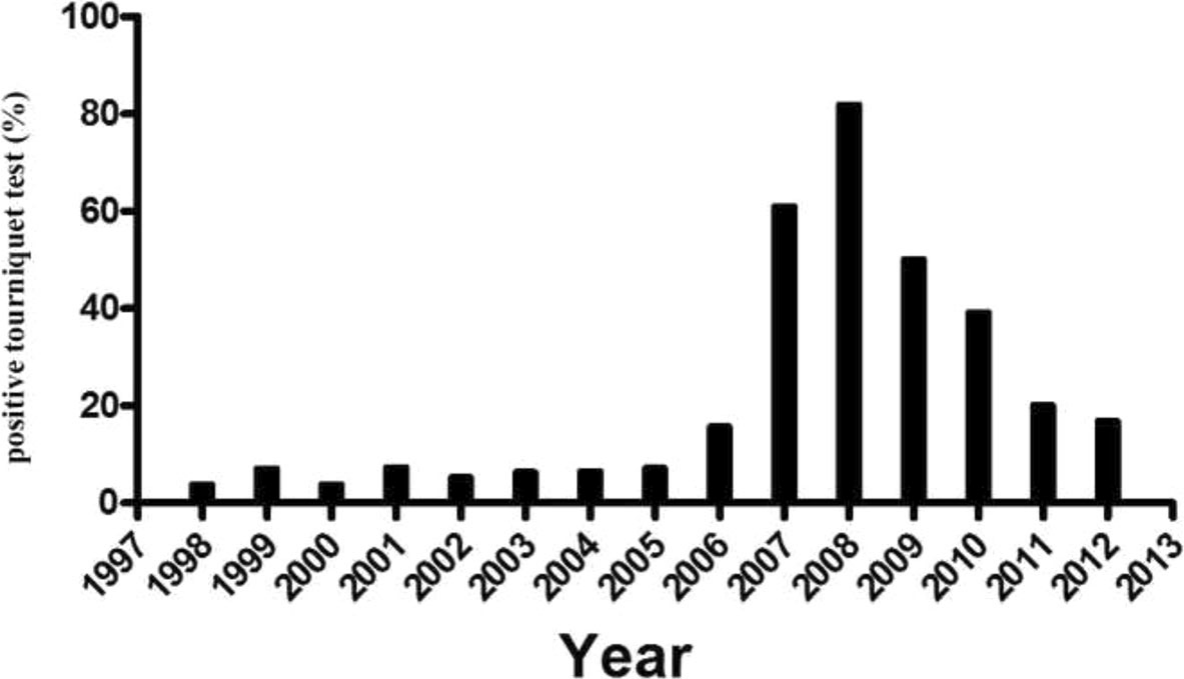
Proportion of positive tourniquet test results between 1998 and 2012 in 25,383 dengue cases

The proportion of positive TT results in patients with dengue over the years shows an increasing trend until 2008, but a decrease in subsequent years (Fig. 1). This ratio was similar within age groups 0 to 14 years (11.2 %), 15 to 60 (12.1 %), and older than 60 years (11.4 %).

Of all cases with TT results, 921 (10.7 %) were dengue without warning signs, 7410 (86.4 %) were dengue with warning signs, 246 (2.9 %) were severe dengue, and 16 patients died. The hypothesis that there would be an association between the TT results and a greater severity score has not been confirmed with the Mann-Whitney test (U = 3,826,121; *P* = 0.28). For this analysis, only 8577 of the 30,670 confirmed cases of dengue had the TT performed, with 1026 (12.0 % of 8577) testing positive and 7551 (88.0 %) testing negative.

The TT had almost no concordance with the final diagnosis on the basis of the Cohen’s Kappa coefficient (κ = 0.01, CI 95 % = 0.00 to 0.02), with IgM detection by ELISA (κ = 0.05; CI 95 % = 0.04 to 0.06), or with PCR (κ = 0.27, CI 95 % = 0.06 to 0.49) (Table 2). The comparison between the TT and the NS1 detection could not be carried out because only four individuals had these two tests performed simultaneously.

**Table 2:** The degree of agreement between the results of the tourniquet test and those of various laboratory methods

	Tourniquet test			
	Positive	Negative	κ ^a^(CI 95 %)	Agreement
	*N* (%)	*N* (%)		
Final diagnosis			0.01 (0.00 to 0.02)	Slight
Positive	1026 (31.5)	7551 (29.7)		
Negative	2226 (68.5)	17,832 (70.3)		
Total	3252 (100.0)	25,383 (100.0)		
ELISA(IgM)			0.05 (0.04 to 0.06)	Slight
Positive	1016 (69.2 %)	7539 (53.4 %)		
Negative	453 (30.8 %)	6590 (46.6 %)		
Total	1469 (100.0)	14,129(100.0)		
PCR			0.27 (0.06 to 0.49)	Fair
Positive	20 (62.5 %)	15 (34.9 %)		
Negative	12(37.5 %)	28 (65.1 %)		
Total	32 (100.0)	43 (100.0)		

Comparison between tourniquet test and NS1 research could not perform because there were only four patients with both tests realized

^a^Cohen’s Kappa coefficient (confidence interval of 95 %)

Based on the final diagnosis obtained, the TT had a sensitivity of 11.9 % (CI 95 % = 0.11 to 0.12), as opposed to a specificity of 88.9 % (CI 95 % = 0.88 to 0.89), indicating an ability to detect truly negative cases. In addition, it was observed that the ability of this test to predict if the patient was actually sick when the test was positive was significantly lower. That is to say, the PPV was only 31.6 % (CI 95 % = 0.31 to 0.32) while the NPV was 70.3 % (CI 95 % = 0.70 to 0.71), showing a greater ability to identify those individuals who really did not have dengue when the TT was negative. However, the positive likelihood ratio (1.08) shows that this does not practically change the chance of the TT results being truly positive in a population with dengue compared to a population without the disease. A similar result was also seen in the negative likelihood ratio (0.99), which showed that the likelihood of a negative test result in patients with dengue was not different from those without the disease.

## Discussion

The approach of the TT value as auxiliary method for diagnosing dengue as presented here is something unusual in the medical literature. The use of so many samples over such a long period of study, and the comparison of the results, including the dengue severity was never reported. About a quarter (28 635) of patients initially notified as suspected dengue fever (119,589) were evaluated by the TT, so a considerable amount of patients were not submitted to this test, perhaps as a consequence of difficulties in filling the notification form or due to non-realization of this test during the great outbreaks of the disease. In our investigation, there was a gradual increase in the percentage of positive TT results over the years, with a further decrease in the last five years of the study, with similar proportions of positive test results between age groups. No association between the TT results and greater severity (according to the 2009 guideline) was observed (*P* = 0.28). The TT results also failed to show relevant agreement with the final diagnosis (κ = 0.01; 95 % CI = 0.00 to 0.02), the ELISA-IgM results (κ = 0.05; 95 % CI = 0.04 to 0.06), and much less with the PCR results (κ = 0.27; 95 % CI = 0.06 to 0.49). This low concordance was also justified by the low sensitivity of the TT to diagnose dengue (11.9 %; 95 % CI = 0.11 to 0.12) and low PPV (31.6 %; 95 % CI = 0.31 to 0.32); nevertheless, it has shown most importance for specificity (88.9 %; 95 % CI = 0.88 to 0.89) and NPV (70.3 %; CI 95 % = 0.70 to 0.71).

The wide clinical spectrum of infection by DENV and the possibility of progression from mild to more severe forms of dengue [30], which require early therapeutic measures and are related to higher mortality, demonstrate the need for alternative methods that can be correlated to this evolution and anticipate potential severity. WHO, in its 1997 guidelines, lists the TT as a criterion in the diagnosis of dengue hemorrhagic fever. In the 2009 guidelines, it is presented as a criterion for both mild and severe forms of the disease. However, studies differ in their use of the test for the diagnosis of infection by DENV and their correlation with severity. Although our findings are in agreement with those found by other authors regarding the TT’s low sensitivity and high specificity [20, 22, 28, 31], there are findings whose values contradict ours, reporting a sensitivity of 83 % and specificity of 23.5 % when used in children in Malaysia [32], suggesting that children may exhibit a different pattern of TT positivity compared to other age groups. However, the finding of equal percentages of positive TT results in both children and adults leads us to disagree with this possibility, despite reports in the literature of different clinical manifestations in children (mainly bleeding) [33]. For other authors, the difference between sensitivity and specificity was practically equal, but with the PPV lower (45 and 55 %, respectively) than the NPV (64 and 69 %, respectively) [27].

Isolated, low PPV was found in our study with a low sensitivity and high specificity; however, Ho et al. [31] reported that PPV may reach 93.1 % when combined with leukopenia (<4000/mm^3^), thrombocytopenia (<150 × 10^3^/mm^3^), partial thromboplastin time (>38 s), elevated aminotransferases (AST/ALT >1.5), and low C-reactive protein (<20 mg/l) [30]. According to Gregory et al. (2011), the combination of a positive TT results and leukopenia (<5000/mm^3^) identified 94 % of patients with dengue in the emergency room [34].

However, leukopenia may not present in the initial stages of dengue, which can delay the diagnosis of the disease when utilizing this method [35]. Therefore, the assessment isolated of the PPV, as well as the NPV, perhaps are not good parameters for evaluation, thus requiring additional laboratory data to make their validation stronger.

It must also be considered that the TT can be influenced by disease progression, age, gender [27], circulating strains, and virus circulation patterns over a determined period. This test can also yield a positive result for other pathological conditions with varying indexes, such as with typhoid fever and Japanese encephalitis [22], although the TT has lower sensitivity for these diseases than for dengue. In this study, 68.5 % of patients who had dengue confirmed by serological diagnosis showed a positive TT result. Understanding this condition could prevent infections with clinical presentations similar to dengue from being misdiagnosed and thus provide a better chance for accurate treatment in adequate time. Such evidence shows the complexity involved in the clinical diagnosis and management of dengue.

Due to the long period of study, the epidemiological conditions of dengue varied, both in relation to the circulating serotype and the number of co-circulating serotypes, which are factors that may be related to a positive TT result. According to the data in Fig. 1, a significant increase in the proportion of positive TTs can be noted until 2008, followed by a decrease. Between the introduction of DENV in the study area and 2008, we observed circulation of DENV-1, −2 and −3, with a maximum of two serotypes simultaneously. Since 2009, there was a reintroduction of DENV-1, followed by explosive outbreaks of DENV-1 and DENV-4 with about 20,000 suspected cases per year and the co-circulation of three serotypes. On the other hand, DENV-3 caused the major epidemics in 2006 and 2007, followed by a DENV-2 epidemic in 2008 [36–39]. Due to insufficient in the database, it was not possible to correlate the data from TTs with serotypes specifically, but the association between serotypes and genotypes may be related to this phenomenon. Moreover, we cannot rule out the possibility that the TT generates a false positive in the presence of other flaviviruses circulating in Brazil, particularly the Saint Louis encephalitis virus, which has been associated with a positive TT in our region [40, 41].

Indeed, in retrospective studies based on government epidemiological data or otherwise, the major limitation is that a lot of information cannot be reassessed, leading to additional reliability being placed on those individuals who collected and recorded information, such as the performance of the TT, which could not be directly supervised. However, it is relatively common that nurses, laboratory technicians, or trained doctors in Brazilian dengue-endemic areas manage dengue cases, which implies that the results are as realistic as possible. Additionally, inconsistent data, both in relation to reporting, such as white blood cell count, as well as the interpretation, were excluded from analysis in this study in order to minimize the effect of these limitations on the findings and conclusions.

## Conclusions

Therefore, the TT was more effective in detecting cases that were truly negative than positive. These results suggest that the TT should not be used as diagnosis of dengue, however, if it is absolutely necessary to use it, as is the case in very poor dengue endemic areas where more sensitive and specific laboratory tests are not available, then it to be done with great caution for screening giving rise to suspicion of dengue cases.

## Abbreviations

ALT: Alanine transaminase
AST: Aspartate transaminase
CI: Confidence interval
DENV: Dengue virus
ELISA: Enzyme-linked immunoassay
FAPESP: Fundação de Amparo à Pesquisa do Estado de São Paulo
NPV: Negative predictive value
NS1: Nonstructural protein 1
PCR: Polymerase chain reaction
PPV: Positive predictive value
SINAN: Notifiable Diseases Information System
TT: Tourniquet test
WHO: World Health Organization
κ: Cohen’s Kappa coefficient
